# Sulfur-Rich Hole-Transporting Materials for Inverted Perovskite Solar Cells

**DOI:** 10.1021/acs.orglett.5c01859

**Published:** 2025-06-19

**Authors:** Yogesh S. Tingare, Lin-Yi Liu, Chaochin Su, Wen-Zheng Lin, Chen-Yi Yen, Wei-Hong Chen, Sheng-Hung Teng, Mei-Jie Chen, Chen-Wei Chu, Wen-Ren Li

**Affiliations:** † Institute of Organic and Polymeric Materials/Research and Development Center for Smart Textile Technology, 34877 National Taipei University of Technology, Taipei 106344, Taiwan; ‡ Department of Chemistry, 34911 National Central University, Zhongli 32001, Taiwan

## Abstract

We introduce an electron-rich hole-transporting material (HTM), **WZ103**, based on a sulfur-rich terthiophene core. This HTM features two triphenylamine donor groups at the 4,4″ positions and four additional triphenylamine groups at the 5,5″ positions connected by vinylene linkages. Its good hole-transporting properties, reduced series resistance, and effective defect passivation contribute to the improved performance. With a remarkable power conversion efficiency of 19.48%, this HTM ranks among the top nonfused terthiophene-based, dopant-free HTMs.

## Graphical abstract

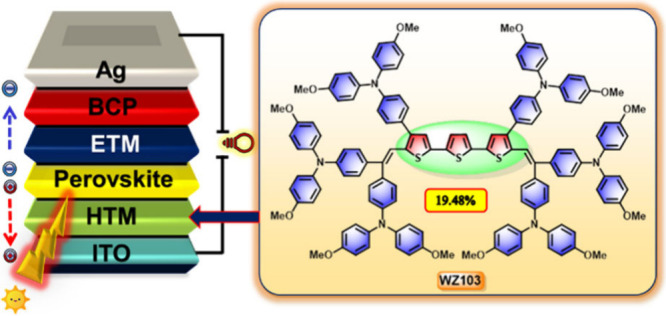

Metal halide perovskite solar cells (PSCs)^1,2^ are revolutionizing photovoltaic technology with a certified power conversion efficiency (PCE) of 26.7%.^3^ Their exceptional properties, high absorption coefficients, charge carrier diffusion lengths, and strong defect tolerance make them ideal for next-generation solar cells.^4–6^ Furthermore, they can be produced cost-effectively, especially in their inverted form.^7–9^ When light interacts with the perovskite layer, it generates electrons and holes that quickly move to the charge-selective layers of hole-transporting materials (HTMs) and electron-transporting material.^10^ The efficiency of PSCs relies heavily on the swift transfer of these charge carriers;^11^ hole transfer occurs in about 1 ns, while electron transfer takes around 11 ns.^12^ This rapid transfer underscores the importance of interfacial charge dynamics for optimal performance.^13^ However, defects in PSCs can lead to electron or hole trapping and nonradiative recombination.^14^ To improve the efficiency of PSCs, it is vital to minimize trap-assisted nonradiative recombination through effective passivation, and the use of passivating interfacial layers can be critical in achieving this goal.^15–17^ Lewis bases containing nitrogen, oxygen, and sulfur are crucial for passivation by interacting with unsaturated iodide and lead ions in perovskite materials.^14,15,18–22^ Incorporating these functional groups into HTMs can enhance defect passivation at the HTM/perovskite interface, preventing ion migration and ensuring stability during perovskite formation.^14,15,18–23^ Innovative building blocks like thiophene, fluorene, carbazole, and pyrene have been developed to create high-performance HTMs.^24–27^ Their successful use in PSCs not only demonstrates their effectiveness but also underscores the critical role of Lewis bases in improving perovskite passivation.^28,29^

Thiophene, a sulfur-rich heterocyclic compound, is an outstanding candidate for HTMs due to its exceptional ability to enhance interactions with perovskite layers.^30,31^ Grimsdale and co-workers pioneered the use of thiophene and bithiophene derivatives in HTMs for PSCs.^32,33^ Grätzel’s group introduced two low-cost thiophene-based HTMs, Z25 and Z26, with Z26 showing a more uniform surface, a higher hole mobility, and a higher conductivity than Z25, underscoring its potential to enhance PSC performance.^34^ Meanwhile, Dai et al. explored the impact of arylamine substitutions on thiophene, revealing that 2,5-substitution notably enhanced π-conjugation for better efficiency.^35^ Li et al. investigated how extending the thiophene chain influences optical properties and hole mobility, finding that longer chains resulted in a red-shift in absorption and improved hole mobility.^36^ Wu et al. assessed thiophene-chain-extended oligomer HTMs in *p-i-n* type PSCs, where the α-quaterthiophene HTM achieved exceptional performance.^37^ Recently, Nazeeruddin et al. introduced innovative HTMs, BT-4D, TT-4D, and QT-4D, finding that BT-4D exhibited outstanding hole extraction properties and long-term stability.^38^ Additionally, while several research groups have explored fused thiophene derivatives, the cost-effective synthesis of nonfused heterocycles indicates that identifying new nonfused ring heterocycle cores for HTMs could be a promising low-cost strategy for developing highly efficient and stable PSCs.^31,39^

We present the design, synthesis, characterization, and photovoltaic performance of a novel sulfur-rich polycyclic aromatic terthiophene core HTM with multiple donor groups. The PCE improves as the number of donor arms on the HTM core increases. In HTM **WZ103**, the core includes two triphenylamine (TPA) donors at the 4,4″ positions and four vinylene TPA groups at the 5,5″ positions (Figure 1). The new terthiophene core design achieved a PCE of 19.48%, making it one of the highest efficiencies reported among inverted devices that utilize nonfused terthiophene-based, dopant-free small molecule HTMs. The high efficiency in **WZ103** is a result of efficient hole transport and reduced series resistance, likely due to the Pb–S interaction occurring at the interface between the terthiophene core and the perovskite.^39^ Such interactions at the perovskite/HTM interface can enhance the operational stability of PSCs and contribute to the overall increase in the PCE.

**Figure 1:**
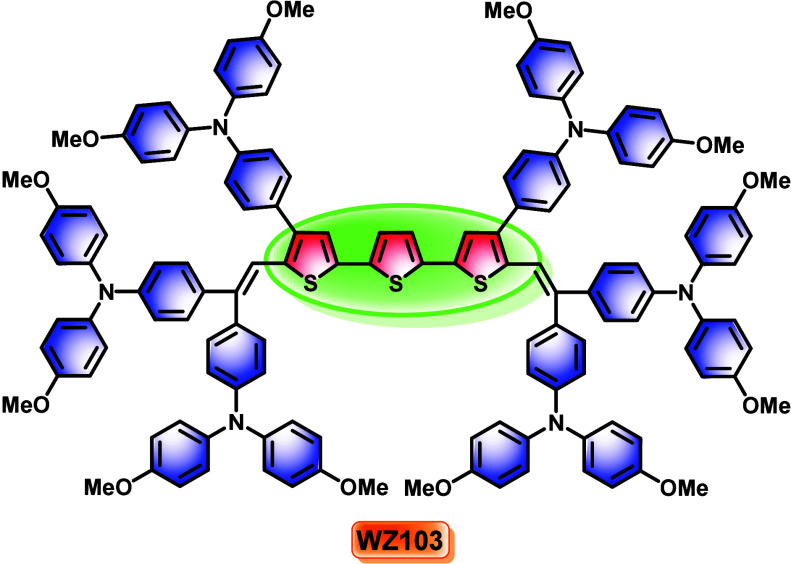
Molecular structure of **WZ103**.

The synthetic route for HTM **WZ103** is illustrated in Scheme 1, and detailed synthetic procedures are shown in the Supporting Information. Initially, the Corey–Fuchs reaction is employed to convert 3-(4-(bis­(4-methoxyphenyl)­amino)­phenyl)­thiophene-2-carbaldehyde (**1**) into an alkyne (**2**).^40^ The metal-catalyzed Suzuki coupling of **2** with (4-(bis­(4-methoxyphenyl)­amino)­phenyl)­boronic acid (**3**) gave compound **4**.^21^ Next, stannylation of **4** in the presence of *n*-BuLi yields the tin compound (**5**). Finally, the metal-catalyzed Stille coupling of compound **5** with 2,5-dibromo thiophene produces final HTM **WZ103**. The thermal properties of HTM **WZ103** were evaluated using thermogravimetric analysis (TGA) (Figure 2a). TGA afforded a thermal decomposition temperature of 421 °C at a 5% weight loss. The absorption spectrum of HTM **WZ103**, recorded in a dichloromethane solution, is shown in Figure 2b, and the data are summarized in Table 1. The absorption spectra of **WZ103** in solution showed a significant peak at 306 nm, attributed to the donor units’ local π–π* electron transition. Additionally, a shoulder peak at approximately 374 nm resulted from the TPA units linked to the terthiophene core. A second high-energy band displayed a notable bathochromic shift, with a peak at 492 nm indicating an intramolecular charge transfer. The electrochemical properties of **WZ103** were analyzed using cyclic voltammetry (Figure S1), as shown in Figure 2c and Table 1. The incorporation of multiple donor moieties increases the highest occupied molecular orbital (HOMO) energy level of **WZ103** to −5.42 eV, which enhances the open-circuit voltage (*V*_OC_) of the PSCs. The favorable HOMO alignment between **WZ103** and MAPb­(I_0.9_Cl_0.1_)_3_ (−5.5 eV) ensures effective hole transport, thereby minimizing nonradiative recombination. The lowest unoccupied molecular orbital energy level was determined to be −3.25 eV, sufficiently high to hinder electron extraction from the perovskite’s conduction band.

**Figure 2:**
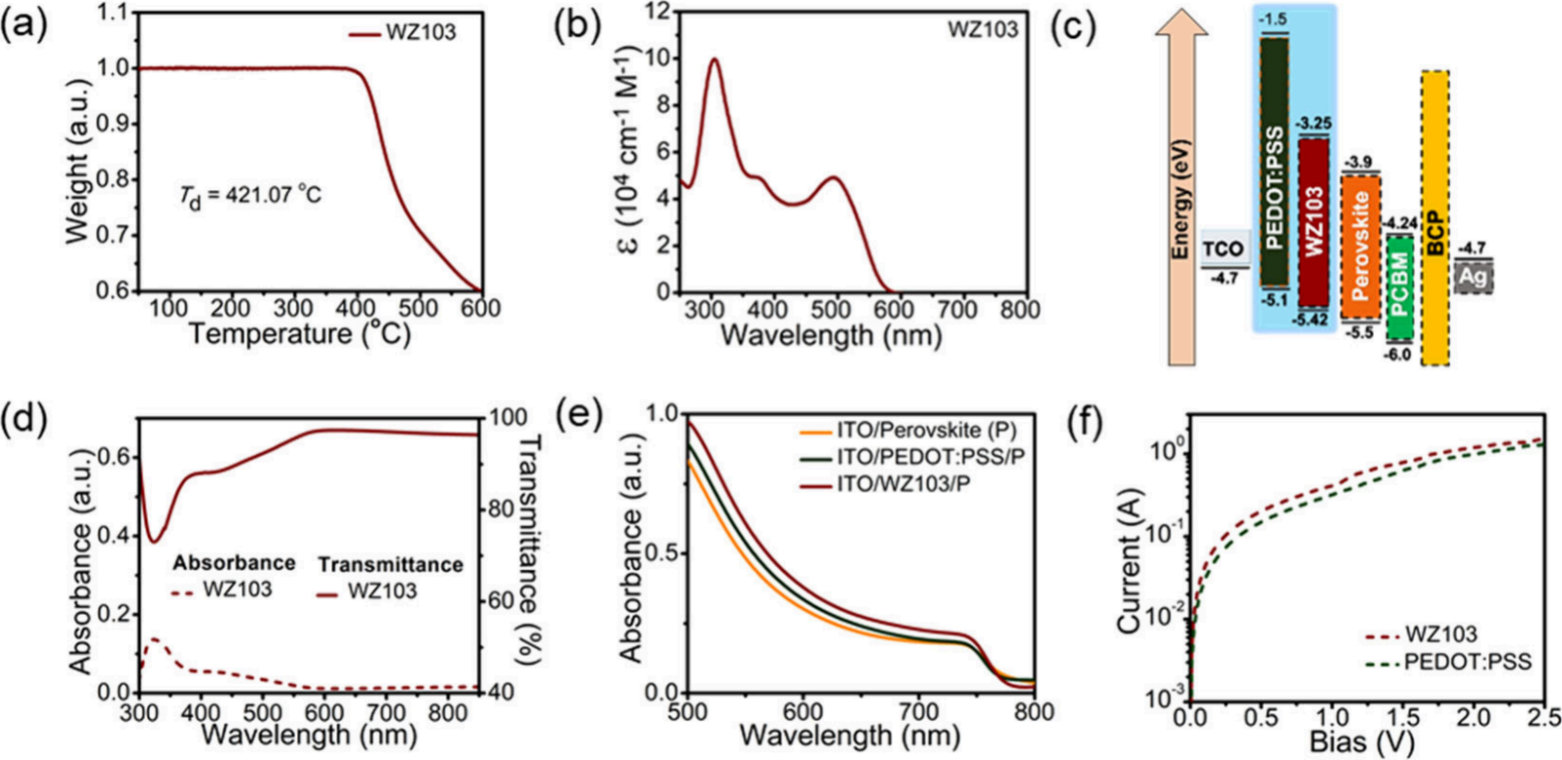
(a) TGA graphs and (b) UV–vis absorption spectra of HTM **WZ103**. (c) Energy levels of PSC devices in this study. (d) UV–vis transmittance spectra of **WZ40** and PEDOT:PSS films. (e) UV–vis spectra of perovskites on different HTMs. (f) SCLC measurement with the ITO/HTM/Au configuration.

**Scheme 1:**
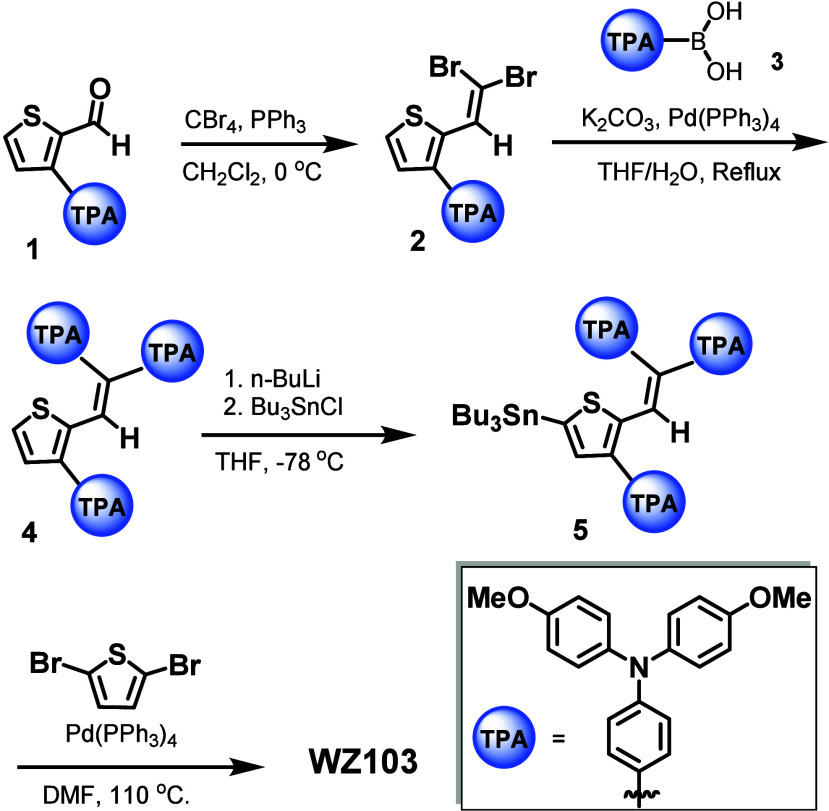
Synthesis of HTM **WZ103**

**Table 1:** Thermal, Optical, and Electrochemical Properties of HTM **WZ103**

λ_max_ ^a^ (nm)	*E*_g_^opt^ (eV)	*E*_HOMO_^b^ (eV)	*E*_LUMO_^c^ (eV)	*T*_d_ (°C)
306, 374, 492	2.17	–5.42	–3.25	421

a Absorption spectra measured in a dichloromethane solution.

b Optical bandgap calculated using the formula 1240/λ onset.

c *E*_LUMO_ = *E*_HOMO_ + *E*_g_^opt^.

High transparency in HTMs is crucial for inverted PSCs, where the perovskite layer is placed above the HTM layer. Our analysis of the UV–visible absorption and transmission of the **WZ103** film, shown in Figure 2d, reveals an impressive transparency with the transmittance exceeding 95% in the 400–800 nm range, ensuring optimal light reaches the perovskite layer. We also measured the UV–visible absorption spectra of perovskite films on HTM **WZ103** and PEDOT:PSS for comparison (Figure 2e). The ITO/**WZ103**/perovskite film displays superior light absorption, indicating that the perovskite over new terthiophene-based HTM **WZ103** efficiently captures the photons. Additionally, we investigated the hole mobilities of **WZ103** by using the space charge limited current (SCLC) technique. As shown in Figure 2f, **WZ103** exhibits a remarkable hole mobility of 1.58 × 10^–4^ cm^2^ V^–1^ s^–1^, outperforming PEDOT:PSS (1.12 × 10^–4^ cm^2^ V^–1^ s^–1^). This enhanced mobility could be attributed to the terthiophene core in **WZ103**, which has a favorable molecular arrangement that improves the charge-transporting properties.

In the inverted architecture, the surface wettability of the HTM plays a crucial role in the quality of perovskite films. Therefore, we measured the contact angles of water droplets on **WZ103** and PEDOT:PSS to evaluate their hydrophobic properties. The results, shown in panels a and b, respectively, of Figure 3, indicated contact angles of 63.19° for **WZ103** and just 13.89° for PEDOT:PSS, highlighting the suitable hydrophobicity of **WZ103**. Panels a and b of Figure 3 display top-view scanning electron microscopy (SEM) images of MAPb­(I_0.9_Cl_0.1_)_3_ films, with cross-sectional SEM images of **WZ103** shown in Figure 3c. The enhanced hydrophobicity of **WZ103** results in films with larger grains and fewer grain boundaries than those on PEDOT:PSS (Figure 3b). Figure 3d shows X-ray diffraction (XRD) patterns of MAPb­(I_0.9_Cl_0.1_)_3_ spin-coated on both surfaces. These patterns reveal sharp peaks for **WZ103**, indicating high purity and crystallinity. Also, atomic force microscopy measurements of **WZ103** and PEDOT:PSS films (Figure S2a,b) conducted over a 2 μm × 2 μm scan area indicate root-mean-square (RMS) roughness values of 6.642 nm for **WZ103**/perovskite and 10.882 nm for PEDOT:PSS/perovskite. These results suggest that the smooth and thin film of **WZ103** is advantageous for producing high-quality polycrystalline perovskite films.

**Figure 3:**
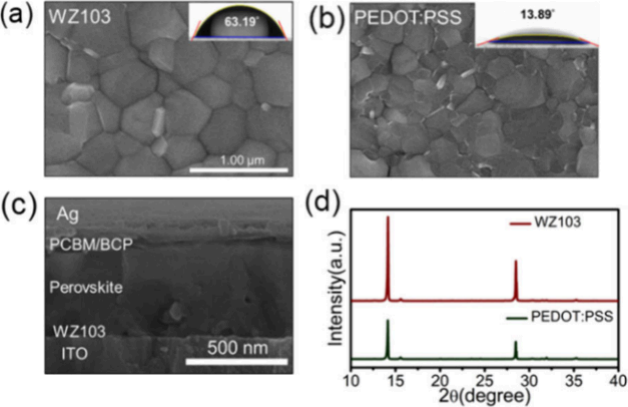
Top-view SEM images of (a) **WZ103** and (b) PEDOT:PSS (contact angles as insets). (c) SEM image of a cross-sectional view of **WZ103**. (d) XRD of perovskite films atop PEDOT:PSS and **WZ103**.

To investigate the performance of terthiophene-based HTM **WZ103**, an inverted PSC was fabricated with the ITO/**WZ103**/MAPb­(I_0.9_Cl_0.1_)_3_/PCBM/BCP/Ag configuration (Figure 4a) and compared to benchmark polymer PEDOT:PSS. Figure 4b displays the current density–voltage (*J*–*V*) curves, and the detailed parameters are presented in Table 2. The results show that a PSC with PEDOT:PSS achieved a *V*_OC_ of 0.95 V, a short-circuit current density (*J*_SC_) of 18.84 mA/cm^2^, a fill factor (FF) of 80.44, and a PCE of 14.36%. In contrast, the PSC utilizing **WZ103** exhibited outstanding results, with a *V*_OC_ of 1.11 V, a *J*_SC_ of 22.96 mA/cm^2^, an FF of 79.20, and a PCE of 19.48%. The enhanced performance of a device based on **WZ103** compared to PEDOT:PSS is due to the higher *V*_OC_ and *J*_SC_. The improvement in *V*_OC_ is attributed to the better alignment with the perovskite’s valence band. The increase in *J*_SC_ results from a reduced number of perovskite defects due to the terthiophene-core Pb–S interaction at the perovskite/HTM interface and the enhanced crystallinity of the perovskite layer, ultimately leading to decreased recombination.

**Figure 4:**
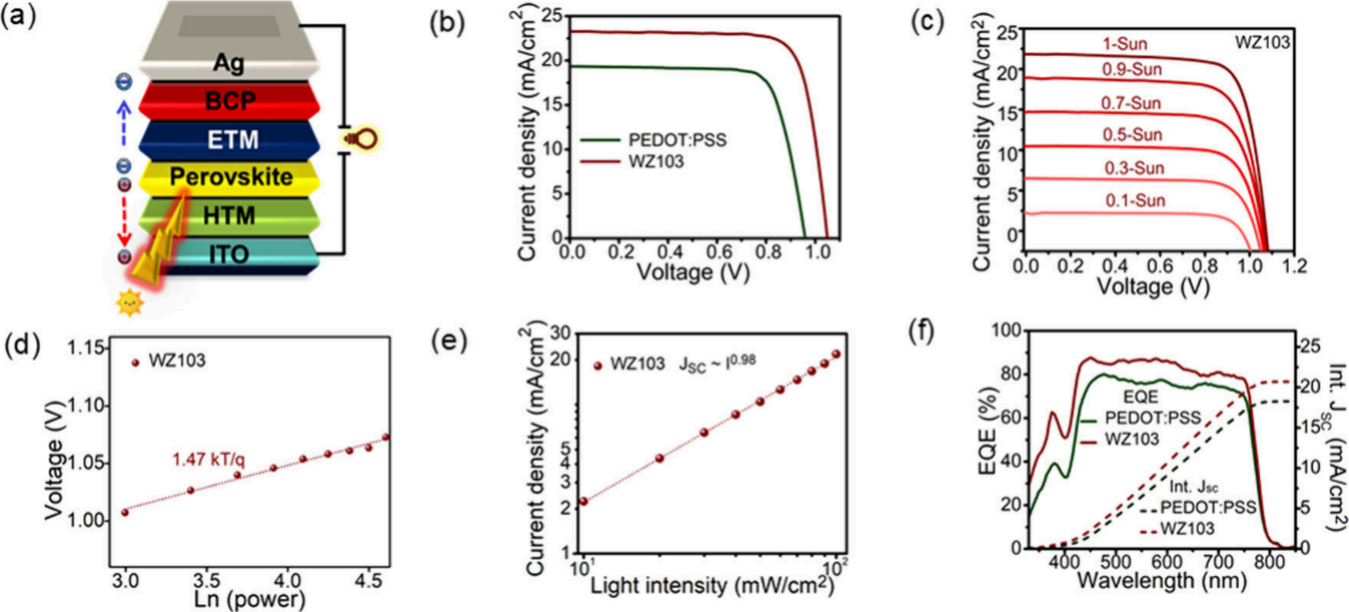
(a) Schematic representation of an inverted PSC device. (b) *J*–*V* curves of **WZ103** and PEDOT:PSS. (c) *J*–*V* curves at various light intensities of **WZ103**. (d) *V*_OC_ and (e) *J*_SC_ as a function of light intensity of the perovskite layer grown on top of **WZ103**. (f) EQE spectra with the integrated *J*_SC_ of the PSCs.

**Table 2:** Photovoltaic Parameters of PSCs Using **WWC103** and PEDOT:PSS

HTM	*V*_OC_ (V)	*J*_SC_ (mA/cm^2^)	FF (%)	η (%)
PEDOT:PSS	0.95	18.84	80.44	14.36
**WZ103**	1.11	22.96	79.20	19.48

Further investigation was conducted using HTM **WZ103** to analyze how illumination intensity impacts the charge recombination mechanism during solar cell operation. Figure 4c shows the characteristic *J*–*V* curve for a perovskite device incorporating **WZ103** across light intensities from 0.1 sun (10 mW/cm^2^) to 1 sun (100 mW/cm^2^). In Figure 4d, the **WZ103** device achieves a slope of 1.47 kT/q in the linear fit of *V*_OC_ versus the logarithm of light intensity. This slope, close to the expected value of 1 kT/q for optimal performance,^22^ indicates reduced trap-assisted recombination, a crucial factor for enhancing efficiency. Additionally, Figure 4e depicts photocurrent variations with light intensity on a logarithmic scale. The nearly linear relationship of *J*_SC_ confirms the efficient retrieval of free carriers without space charge interference. The power parameter of 0.98 for **WZ103** further emphasizes this efficiency. Finally, Figure 4f presents the external quantum efficiency (EQE) and calculated *J*_SC_ for devices using **WZ103** and PEDOT:PSS HTMs. The EQE spectrum (Figure 4f) of the PSC using **WZ103** demonstrates a high conversion efficiency across the entire wavelength range of 300–800 nm. The integrated current densities calculated from the EQE spectra for **WZ103** and PEDOT:PSS are 20.74 and 18.32 mA/cm^2^, respectively.

To validate the performance of **WZ103**, we measured the stabilized photocurrent density, as shown in Figure S3a. The results indicate an impressive stabilized *J*_SC_ of 20.68 mA/cm^2^, with a minimal decrease over 1000 s, highlighting the device’s effectiveness under the tested conditions. We also assessed the solar cell’s long-term stability at 25 and 70 °C using **WZ103**, showing remarkable stability by retaining 81% and 71% of their original PCE after 500 and 200 h, respectively (Figure S3b,c). Additionally, we performed steady-state photoluminescence (PL) measurements (Figures S3d). The PL analysis reveals that the pristine perovskite film on ITO glass shows the strongest PL response, which significantly decreases for perovskite films on various HTMs. The PL quenching order is as follows: **WZ103**/perovskite > PEDOT:PSS/perovskite > pristine perovskite. This indicates that **WZ103** has superior hole extraction capability compared to PEDOT:PSS.

In conclusion, we have developed an innovative dopant-free HTM with multiple peripheral donors and a terthiophene central core. The strong interactions between sulfur and lead at the terthiophene core of **WZ103** result in high-quality perovskite films with passivated interface trap states, improving stability and sustainability. Additionally, the well-aligned energy levels facilitate efficient charge extraction, greatly boosting device performance. Light intensity studies support these findings, revealing that devices using **WZ103** exhibit a superior linear response. As a dopant-free HTM in inverted PSCs, it achieved an impressive efficiency of more than 19.48%, surpassing that of PEDOT:PSS (14.36%). In summary, this molecular design enhances the perovskite/HTM interface, reduces charge recombination, and optimizes hole mobility, leading to greater stability and high-performance PSCs.

## Supplementary Material



## Data Availability

The data underlying this study are available in the published article and its Supporting Information.

## Notes

Synthetic procedures and details of the preparation and characterization of PSC devices (PDF)
