# Supplementary material for: Sulfur-Rich Hole-Transporting Materials for Inverted Perovskite Solar Cells
Source: Org Lett. 2025 Jun 19;27(26):6983–8. doi: 10.1021/acs.orglett.5c01859 (PMC12235625; doi:10.1021/acs.orglett.5c01859)
Supplement: Supplementary file 1 [file ol5c01859_si_001.pdf]

Supporting Information

Sulfur-rich Hole-transporting Materials for Inverted Perovskite Solar Cells

Yogesh S. Tingare[a,], Lin-Yi Liu[a], Chaochin Su[a,*], Wen-Zheng Lin[b], Chen-Yi Yen[b], Wei-Hong Chen[a], Sheng-Hung Teng[b], Mei-Jie Chen[a], Chen-Wei Chu[a], and Wen-Ren Li[b,*]*

[a]Prof. Y. S. Tingare, Prof. C. Su,

Institute of Organic and Polymeric Materials/Research and Development Center for Smart Textile Technology

National Taipei University of Technology

Taipei 106344, Taiwan

E-mail: f12098@ntut.edu.tw; f10913@mail.ntut.edu.tw

[b]Prof. W.-R. Li

Department of Chemistry

National Central University

Zhongli 32001, Taiwan

E-mail: ch01@ncu.edu.tw

Materials: A silica gel (Scharlau 60, 230-240 mesh) was used for flash chromatography. Thin-layer chromatography (TLC) was used to monitor the reaction progress using aluminum-coated Merck Kieselgel 60 F254 plates developed with I_2 or UV light. All intermediates and final products were analyzed using 1H and 13C NMR spectra obtained by a Bruker Avance 500 MHz spectrometer. Chemical shifts (δ) and coupling constants (*J*) are expressed in Hz and ppm, respectively. Multiplicities were reported as s = singlet, d = doublet, t = triplet, q = quartet, p = pentet, m = multiplet, and b = broad. Mass spectra matrix-assisted laser desorption ionization-time-of-flight (MALDI-TOF) experiments were recorded using a Bruker Daltonics flex Analysis spectrometer. CV experiments were conducted using a CHI 627C electrochemical setup with a glassy carbon electrode as the working electrode, a silver wire ($Ag/AgNO_3$ in acetonitrile) as the reference electrode, a Pt wire as the counter-electrode, and 0.1 M tetrabutylammonium hexafluorophosphate as the supporting electrolyte. The cyclic voltammograms of HTMs were typically recorded at a scan rate of 50 mVs^{-1}. Following the measurement, ferrocene was used as an internal reference for calibration. Thermogravimetric analysis (TGA) was performed using a TA Instruments with a ramp rate of 10 ˚C/min under N_2 from 100 to 600 ˚C. A Shimadzu UV-vis-NIR spectrophotometer was used to examine the films' optical characteristics. Scanning electron microscopy (SEM) and X-ray diffraction (XRD) using a BRUKER ECO D8 series were utilized to examine the morphology of the perovskite films. A spectrofluorometer (FS5; Edinburgh Instruments) was used to measure time-resolved photoluminescence (PL) spectra with the PL

excitation set to 405 nm. The contact angle was measured using a Phoenix 300 from Surface Electro-Optics (SEO) Co., Ltd. with de-ionized water as the solvent.

EXPRIMENT SECTION

Synthesis of 4-(2-(2,2-dibromovinyl)thiophen-3-yl)-N,N-bis(4-methoxyphenyl)aniline (2): In a 50 mL flame-dried round bottom flask, a solution of CBr_4 (1.59 g, 4.81 mmol) in dry CH_2Cl_2 (4.3 mL, 0.56 M) was cooled to 0 ˚C in an ice bath. PPh_3 (2.53 g, 9.63 mmol) was then added slowly at 0 ˚C, and the reaction was stirred for 10 minutes. A solution of compound 9 (6.03 mL, 0.4M) in dry CH_2Cl_2 was added slowly at 0 ˚C to the reaction mixture. The reaction was stirred at 0 ˚C for 10 minutes, then at room temperature for 1 hour. The mixture was then extracted with CH_2Cl_2 and washed with water. The organic layer was dried over $MgSO_4$ and evaporated under reduced pressure. The crude product was purified by column chromatography (ethyl acetate/hexane = 10%) to yield compound **2** (1.08 g, 78.4%) as a bright yellow solid. ^{1}H NMR (500 MHz, $(CD_3)_2SO$): δ 7.74 (d, *J* = 5.0 Hz, 1H), 7.57 (s, 1H), 7.18 (d, *J* = 8.0 Hz, 2H), 7.15 (d, *J* = 5.5 Hz, 1H), 7.10 (d, *J* = 8.5 Hz, 4H), 6.93 (d, *J* = 8.5 Hz, 4H), 6.78 (d, *J* = 8.0 Hz, 2H), 3.74 (s, 6H); ^{13}C NMR (125 MHz, $(CD_3)_2SO$): δ 156.1, 148.2, 143.9, 139.5, 130.1, 130.0, 129.7, 128.81, 127.4, 127.2, 126.0, 118.1, 115.0, 87.1, 55.2; HRMS (MALDI-TOF) calcd for $C_{26}H_{21}Br_2NO_2S$ $[M]^+$: 568.9660, found 568.9654.

Synthesis of 4,4'-(2-(3-(4-(bis(4-methoxyphenyl)amino)phenyl)thiophen-2-yl)ethene-1,1-diyl)bis(N,N-bis(4-methoxyphenyl)aniline) (4): A mixture of compound **2** (1.00 g, 1.75 mmol), (4-(bis(4-methoxyphenyl)amino)phenyl)boronic acid (**3**) (2.26 g, 5.25 mmole), $Pd(PPh_3)_4$ (101.3 mg, 0.09 mmol), and potassium carbonate (1.45 g, 10.50 mmol) in DMF/H_2O (4/1) was refluxed using an oil bath under an argon atmosphere for 6 hours. After cooling down the reaction to room temperature, the mixture was extracted with ethyl acetate and washed with water. The organic layer was collected and evaporated under reduced pressure. The crude product was purified by column chromatography (ethyl acetate/hexane = 20%) to afford compound **4** (0.90 g, 50.1 %) as a yellow solid ^{1}H NMR (500 MHz, $(CD_3)_2SO$): δ 7.29 (d, *J* = 5.0 Hz, 1H), 7.19 (d, *J* = 8.5, 2H), 7.05 (d, *J* = 9.0 Hz, 4H), 7.01 (d, *J* = 9.0 Hz, 4H), 6.96-6.88 (m, 14H), 6.86 (d, *J* = 9.0 Hz, 4H), 6.84 (d, *J* = 9.0 Hz, 4H), 6.80 (d, *J* = 8.5 Hz, 2H), 6.73 (d, *J* = 8.5 Hz, 2H), 6.60 (d, *J* = 9.0 Hz, 2H), 3.72 (s, 6H), 3.70 (s, 6H), 3.69 (s, 6H); ^{13}C NMR (125 MHz, $(CD_3)_2SO$): δ 155.8, 155.7, 155.6, 148.1, 147.7, 147.4, 141.2, 140.0, 139.8, 139.8, 139.7, 134.8, 133.5, 131.0, 130.5, 129.7, 127.9, 127.7, 127.4, 126.8, 126.7, 126.5, 125.6, 119.8, 118.7, 118.6, 117.5, 114.9, 114.8, 55.1; HRMS (MALDI-TOF) calcd for $C_{66}H_{57}N_3O_6S$ $[M]^+$: 1019.3968, found 1019.3963.

Synthesis of 4,4'-(2-(3-(4-(bis(4-methoxyphenyl)amino)phenyl)-5-(tributylstannyl)thiophen-2-yl)ethene-1,1-diyl)bis(N,N-bis(4-methoxyphenyl)aniline) (5): In a 50 mL flame-dried round bottom flask, compound **4** (0.88 g, 0.850 mmol) was dissolved in dry THF (4.3 mL, 0.2 M), and then *n*-BuLi (2.5 M in hexane, 0.44 mL, 1.10 mmol) was added dropwise at -78 °C. After stirring for 1 hour, tributyltin chloride (0.3 mL, 1.10 mmol) was added while stirring the solution at -78

°C. The reaction mixture was allowed to warm to room temperature and stirred for 5 hours. After quenching with a small amount of water, the resulting mixture was concentrated and diluted with ethyl acetate. The solution was washed with brine and water, and the aqueous phase was extracted twice with ethyl acetate. The combined organic layer was dried over $MgSO_4$ and evaporated under reduced pressure. The crude product was purified by column chromatography (using neutralized silica gel) with a mixture of ethyl acetate and hexane (15%), resulting in the isolation of compound **5** (0.88 g, 79%) as a light-yellow oil. ^1H NMR (500 MHz, $(CD_3)_2SO$): δ 7.18 (d, *J* = 8.5 Hz, 2H), 7.01-7.00 (m, 8H), 6.96-6.92 (m, 10H), 6.87-6.81 (m, 14H), 6.75 (d, *J* = 8.5 Hz, 2H), 6.61 (d, *J* = 8.5 Hz, 2H), 3.71 (s, 6H), 3.70 (s, 6H), 3.69 (s, 6H), 1.55-1.39 (m, 6H), 1.28-1.20 (m, 6H), 1.09-0.95 (m, 6H), 0.79 (t, *J* = 7.5 Hz, 9H); ^{13}C NMR (125 MHz, $(CD_3)_2SO$): δ 155.8, 155.5, 147.9, 147.6, 147.3, 142.3, 140.8, 140.2, 139.9, 139.8, 139.5, 136.3, 136.1, 133.6, 131.0, 129.6, 128.0, 127.3, 126.7, 126.6, 126.2, 120.2, 118.9, 118.8, 117.5, 114.9, 114.7, 55.2, 28.4, 26.4, 13.4, 10.4.

Synthesis of final HTM WZ103: In a 5mL round bottom flask, compound **5** (0.88 g, 0.67 mmol), 2,5-dibromothiophene (70 mg, 0.29 mmol), and $Pd(PPh_3)_4$(16.94 mg, 0.02 mmol) were dissolved in DMF (2.3 mL, 0.2 M). The mixture was refluxed at 110 °C using an oil bath under an argon for 12 hours. After cooling to room temperature, the reaction mixture was extracted with CH_2Cl_2, and the organic layer was washed with water. The combined organic layer was dried over $MgSO_4$ and evaporated under reduced pressure. The crude product was purified by column chromatography (ethyl acetate/CH_2Cl_2/hexane = 10/30/60%) to yield HTM **WZ103** (0.61 g, 97.4%) as a red solid. ^1H NMR (500 MHz, $(CD_3)_2SO$): δ 7.24 (d, *J* = 8.0 Hz, 2H), 7.16 (d, *J* = 13.0 Hz, 2H), 7.06-7.00

(m, 16H), 6.96-6.92 (m, 3H), 6.91-6.88 (m, 9H), 6.80 (d, J = 9.0 Hz, 4H), 6.77 (d, J = 8.5 Hz, 2H), 6.66 (d, J = 9.0 Hz, 2H), 3.73 (s, 6H), 3.72 (s, 6H), 3.60 (s, 6H); ^{13}C NMR (125 MHz, $(CD_3)_2SO$): δ 155.8, 155.8, 155.3, 148.4, 147.8, 147.6, 142.2, 140.3, 140.1, 139.7, 139.6, 135.3, 134.5, 134.25, 132.7, 132.7, 131.2, 130.9, 129.6, 127.4, 126.8, 126.7, 125.7, 124.5, 124.2, 121.6, 118.6, 118.4, 117.1, 114.8, 114.7, 55.1, 54.9; HRMS (MALDI-TOF) calcd for $C_{136}H_{114}N_6O_{12}S_3$ $[M]^+$: 2118.7657, found 2118.7651.

Device fabrication and characterizations: The indium tin oxide (ITO) conducting glass substrates were cleaned in an ultrasonic cleaner for 15 minutes using detergent, deionized water, acetone, and isopropanol. After washing, the substrates were exposed to UV-ozone for 15 minutes and then transferred to a glovebox filled with nitrogen. Inside the glovebox, **WZ103** (concentration 2.5 mg/mL) was prepared using anhydrous chlorobenzene solvent. Next, 50 μL of each solution was spin-coated onto the ITO substrates at a rate of 3000 rpm for 30 seconds and then annealed at 110 ˚C for 30 minutes. The **PEDOT:PSS** stock solution was filtered through a 0.22 μm PVDF filter before being spin-coated onto the ITO at 3500 rpm for 40 seconds and heated at 110 ˚C for 30 minutes.

To prepare the mixed-halide perovskites ($MAPb(I_{0.9}Cl_{0.1})_3$ solution), MAI (206.65 mg), PbI_2 (509.52 mg), and $PbCl_2$ (54.23 mg) were mixed in 1000 μL of DMF:DMSO (885:1115) solvent and stirred at 70 ˚C inside the N_2-filled glovebox. The resulting perovskite solution (50 μL) was then spin-coated onto the HTM layers at a rate of 2700 rpm for 50 seconds, and 200 μL of toluene

was dropped onto the perovskite layer at 35 seconds before the end. After dropping the antisolvent, a 5 psi N_2 gas stream was blown through the film and left for 30 seconds as part of the gas-assisted method. The films were annealed at 60 ˚C for 5 minutes and then at 100 ˚C for 3 minutes.

After cooling, 50 μL of [6,6]-phenyl-C_{61}-butyric acid methyl ester (PCBM, 20 mg mL^{-1} in chlorobenzene), filtered through a 0.45 μm PTFE filter, was spin-coated at 2000 rpm for 40 seconds and annealed at 90 ˚C for 5 minutes. Subsequently, the film was cooled, and 60 μL of BCP (0.5 mg mL^{-1} in IPA) was spin-coated at 4000 rpm for 50 seconds. Finally, a 100 nm thick Ag electrode was deposited by thermal evaporation in a vacuum deposition chamber with a 10^{-7} torr pressure.

Device characterizations:

The J-V characteristics were measured using Keithley 2400 measurement source units, kept at room temperature in a glovebox. Photovoltaic testing was performed at 100 mW/cm² using a calibrated solar simulator (Class 3A, SS-F5-ABA, Enli Technology). To calibrate the light intensity, a standard photovoltaic reference cell (SRC-2020 series, Enli Technology) was used. Additionally, Enli Technology's software (IVS-KA6000) was used to modify the light intensity test. The EQE spectrum was measured using Enli Technology's QE-R Model.

Table S1: Parameters of different thiophene based HTMs in Planer PSCs.

HTMs	V_{oc} (V)	J_{sc} (mA/cm^{-2})	*FF*	η (%)	Ref.
H101	1.05	19.1	65	13.2	[1]
H111	1.08	19.8	72	15.4	[2]
H112	1.07	20.0	71	15.2	[2]
H-3,4	0.88	9.34	20	1.67	[3]
H-2,5	0.91	19.46	36	6.40	[3]
KTM-3	0.99	10.3	70.9	7.3	[4]
KM03	1.00	15.72	44.9	7.08	[5]
KM05	0.99	21.40	67.9	14.44	[5]
KM07	1.04	19.34	66	13.30	[5]
M104	1.03	21.01	70.5	15.37	[5]
Z25	1.14	23.12	64	16.9	[6]
Z26	1.13	23.59	75	20.1	[6]
BT-4D	1.05	23.21	78.9	19.34	[7]
TT-4D	1.04	22.77	71.8	17.04	[7]
QT-4D	1.05	22.39	70.3	16.55	[7]
V862	1.13	22.5	77	19.96	[8]
M03	1.07	20.83	69	15.37	[9]
M04	1.03	22.57	71	16.50	[9]
TTB1	0.84	4.11	61.6	2.13	[10]
TTB2	0.97	8.67	72.7	6.12	[10]
TTB3	0.97	10.92	66.07	7.03	[10]
D1	1.12	22.07	66	16.5	[11]
EDOT-OMeTPA	0.95	18.9	61	11.0	[12]

Table S2: Parameters of different thiophene based HTMs in inverted PSCs.

**HTMs**	V_{oc} **(V)**	J_{sc} **(mA/cm^{-2})**	***FF***	**η (%)**	**Ref.**
**BT**	1.00	19.50	78.1	15.21	[13]
**QT**	1.03	21.05	81.5	17.69	[13]
**HT**	1.03	18.10	72.1	13.47	[13]
**WZ103**	1.11	22.96	79.20	19.48	This work

Table S3: Parameters of **WZ103**-Based Inverted PSC exposed to different humidity.

**WZ103**	V_{oc} **(V)**	J_{sc} **(mA/cm^{-2})**	***FF***	**η (%)**
0% RH	1.03±0.01	21.06±0.21	71.82±0.11	15.64±0.09
15% RH	1.03±0.02	21.07±0.16	74.68±0.21	16.21±0.14
25% RH	1.02±0.02	21.28±0.15	75.52±0.71	16.43±0.05
35% RH	1.04±0.02	21.98±0.22	71.82±0.11	16.36±0.17
45% RH	1.05±0.01	23.11±0.19	67.32±0.18	16.34±0.13

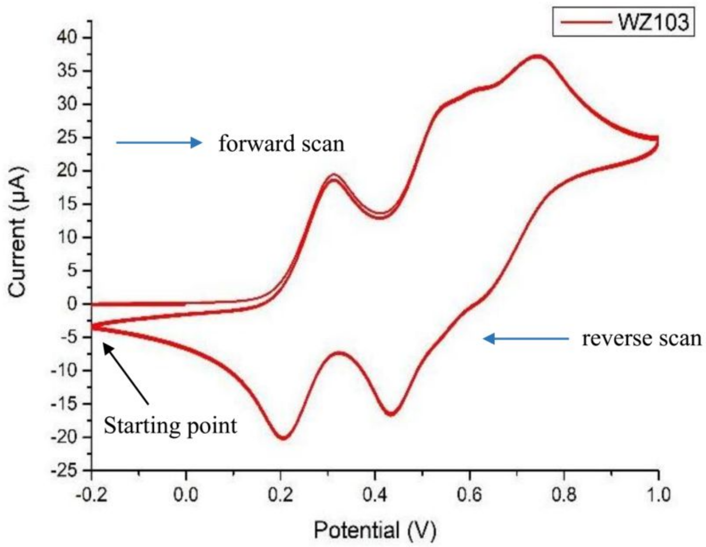

Figure S1. Cyclic voltammogram recorded using a glassy carbon working electrode (50 mm diameter, geometric surface area =19.63 cm²), platinum wire counter electrode, and Ag/$AgNO_3$ reference electrode in dry dichloromethane (DCM). Ferrocene was added as an external standard and all potentials were corrected to ferrocene. The electrolyte solution contained 0.1 M tetrabutylammonium hexafluorophosphate ($TBAPF_6$) and 1.0×10^{-3} M analyte. The scan was conducted at a rate of 0.05 V s^{-1} at room temperature. The potential is plotted according to IUPAC convention, increasing from left (negative) to right (positive).

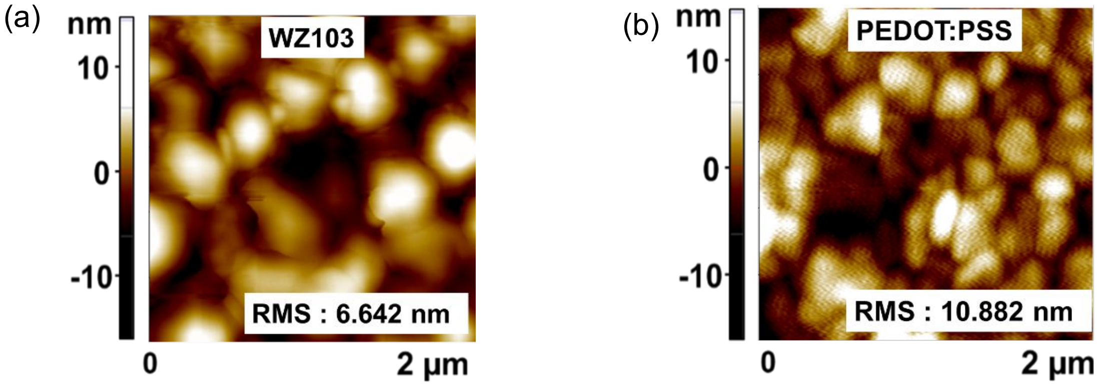

Figure S2. AFM images (2μm × 2μm) of (a) **WZ103** and (b) **PEDOT:PSS**.

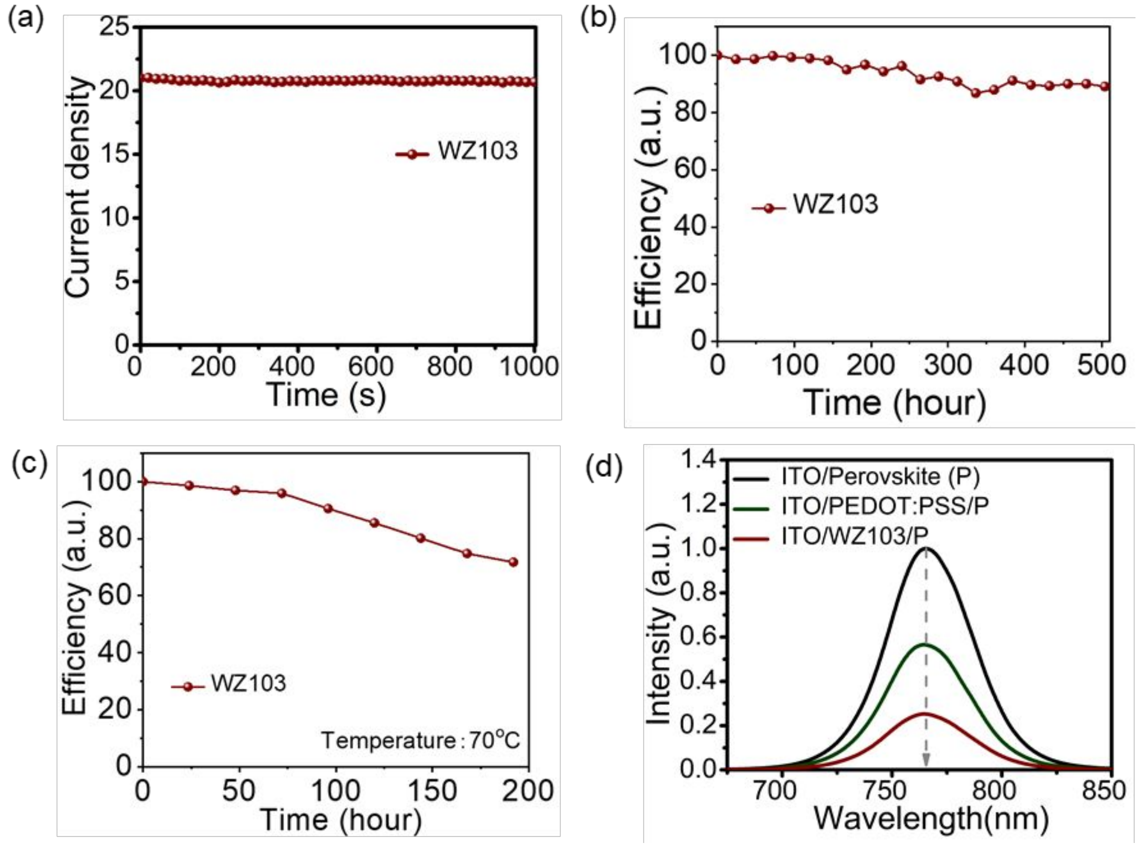

Figure S3. (a) Steady-state photocurrent output of PSCs with **WZ103** HTM. The stability testing of the PSCs incorporated with **WZ103** (b) measured at 25 °C (c) measured at 70 °C under nitrogen atmosphere. (d) Steady-state PL for perovskites and perovskites/HTM interface.

^{1}H and ^{13}C NMR

Solvent: $(CD_3)_2SO$

NMR Hz: 500 MHz

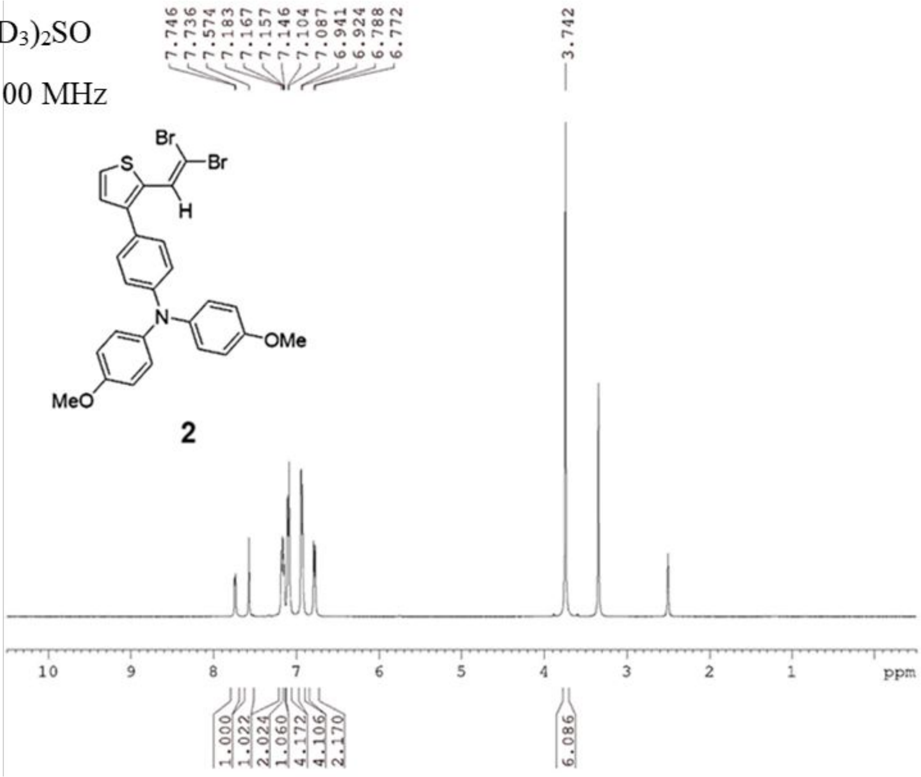

Solvent: $(CD_3)_2SO$

NMR Hz: 125 MHz

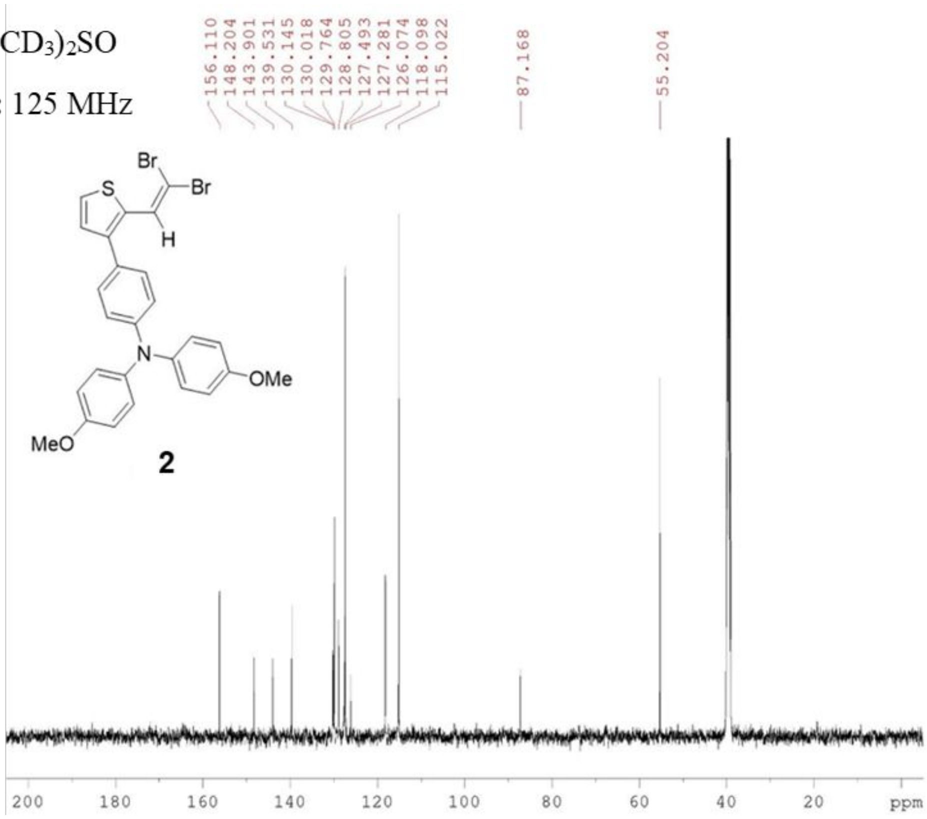

Solvent: $(CD_3)_2SO$

NMR Hz: 500 MHz

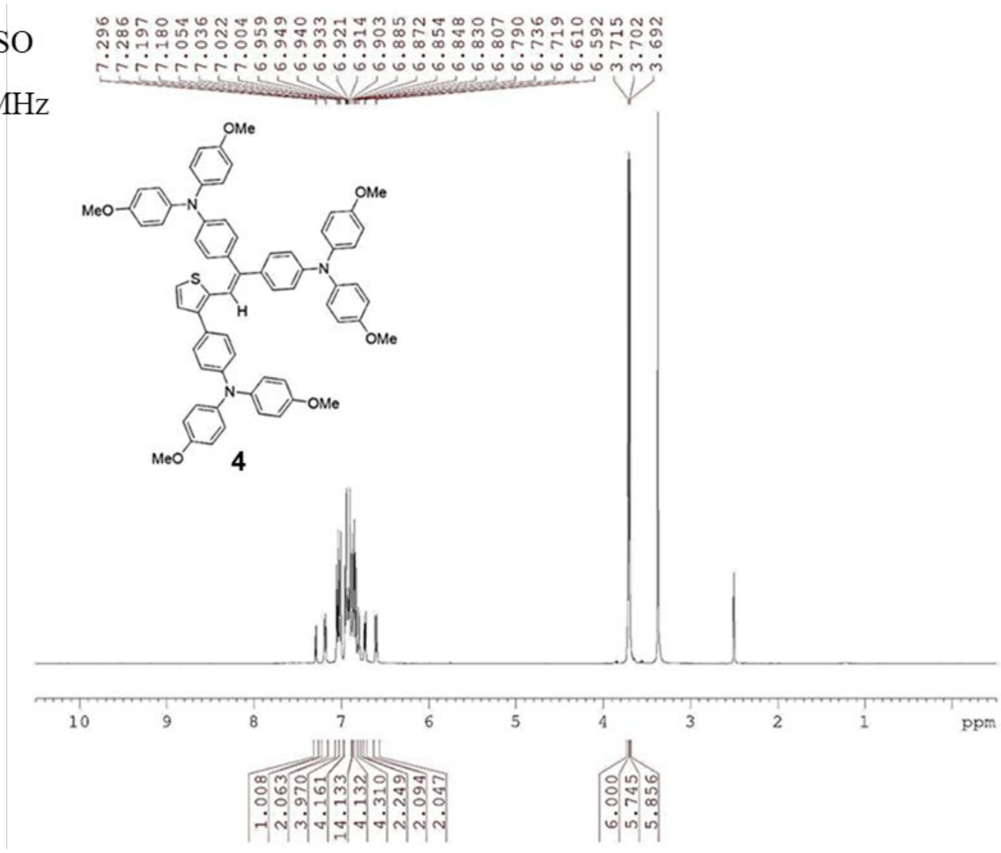

Solvent: $(CD_3)_2SO$

NMR Hz: 125 MHz

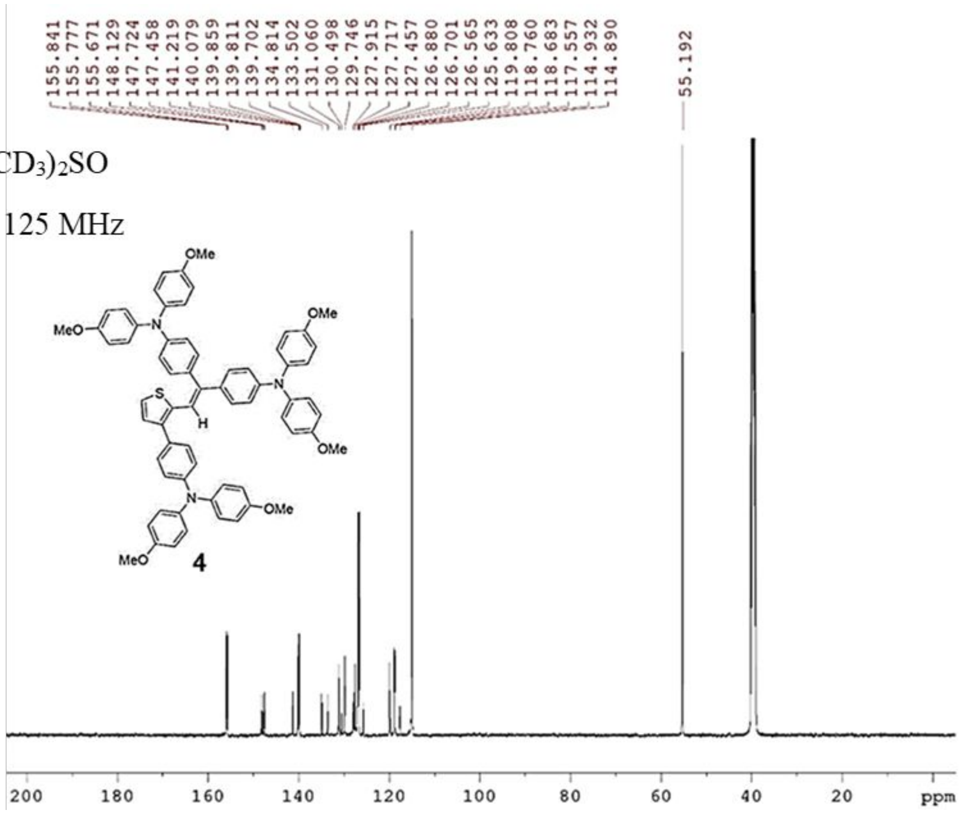

Solvent: $(CD_3)_2SO$

NMR Hz: 500 MHz

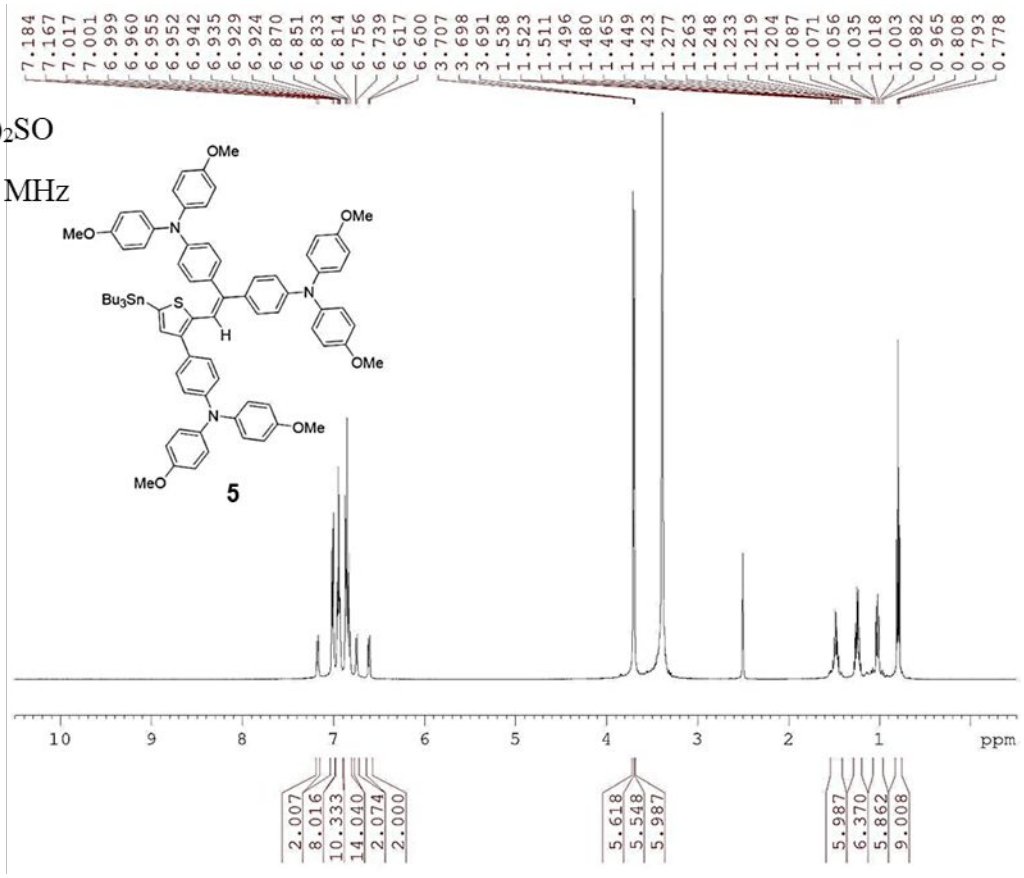

Solvent: $(CD_3)_2SO$

NMR Hz: 125 MHz

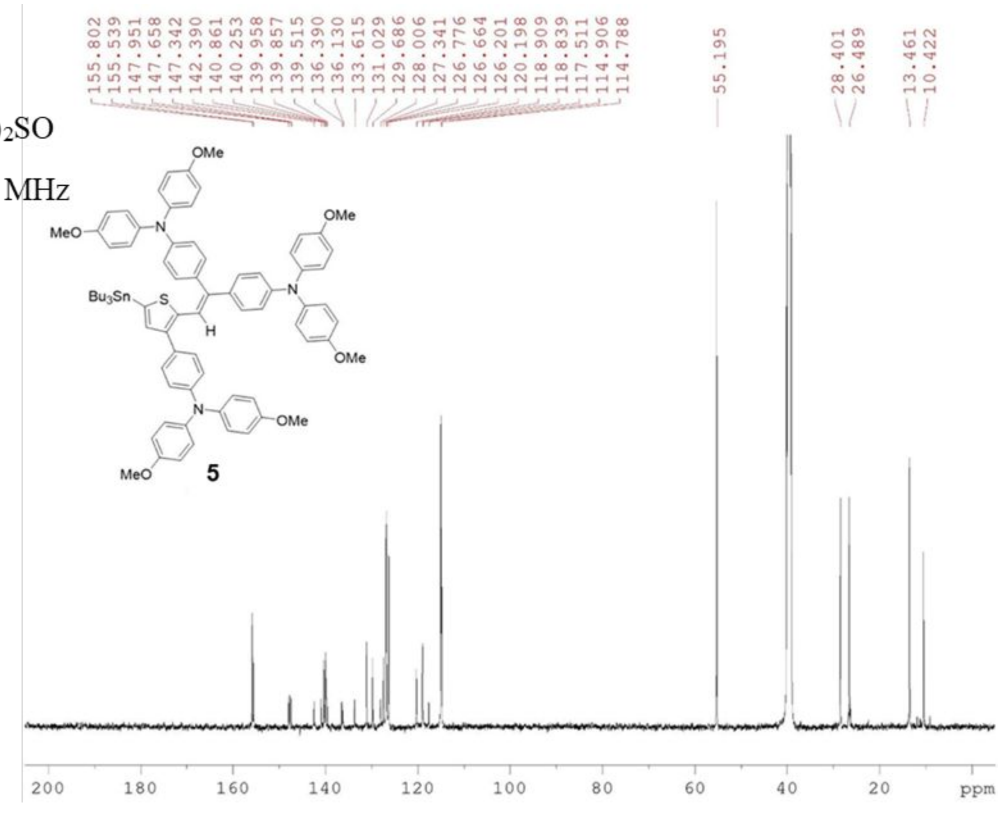

Solvent: $(CD_3)_2SO$

NMR Hz: 500 MHz

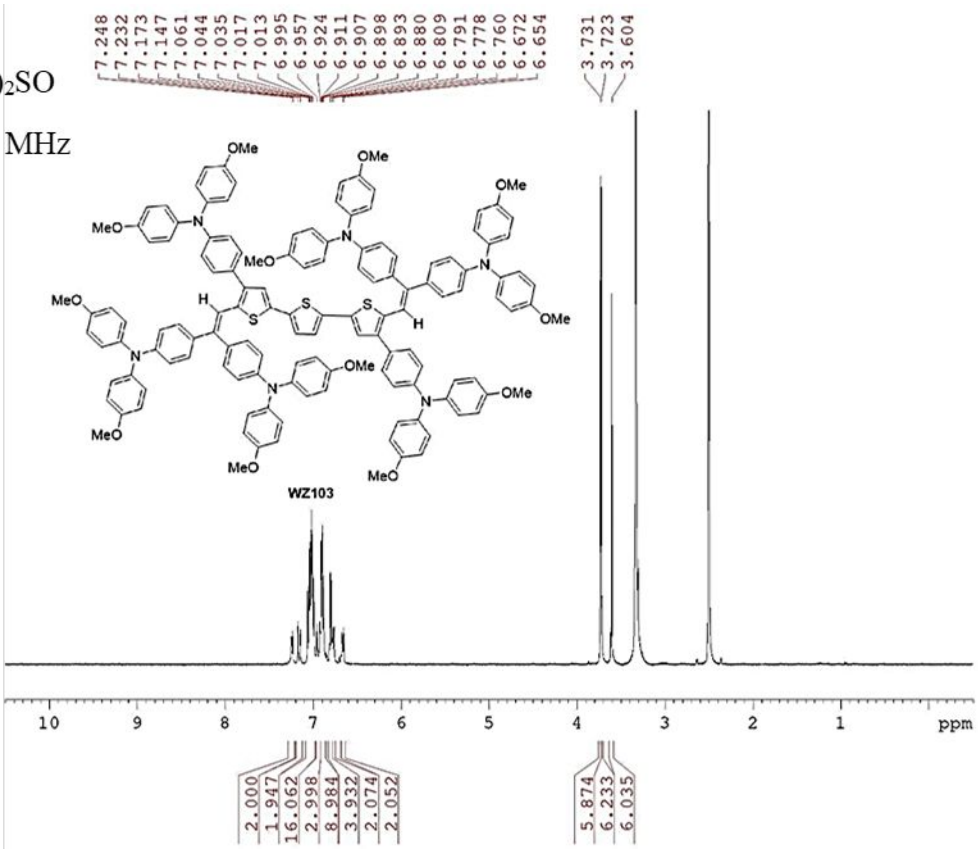

Solvent: $(CD_3)_2SO$

NMR Hz: 125 MHz

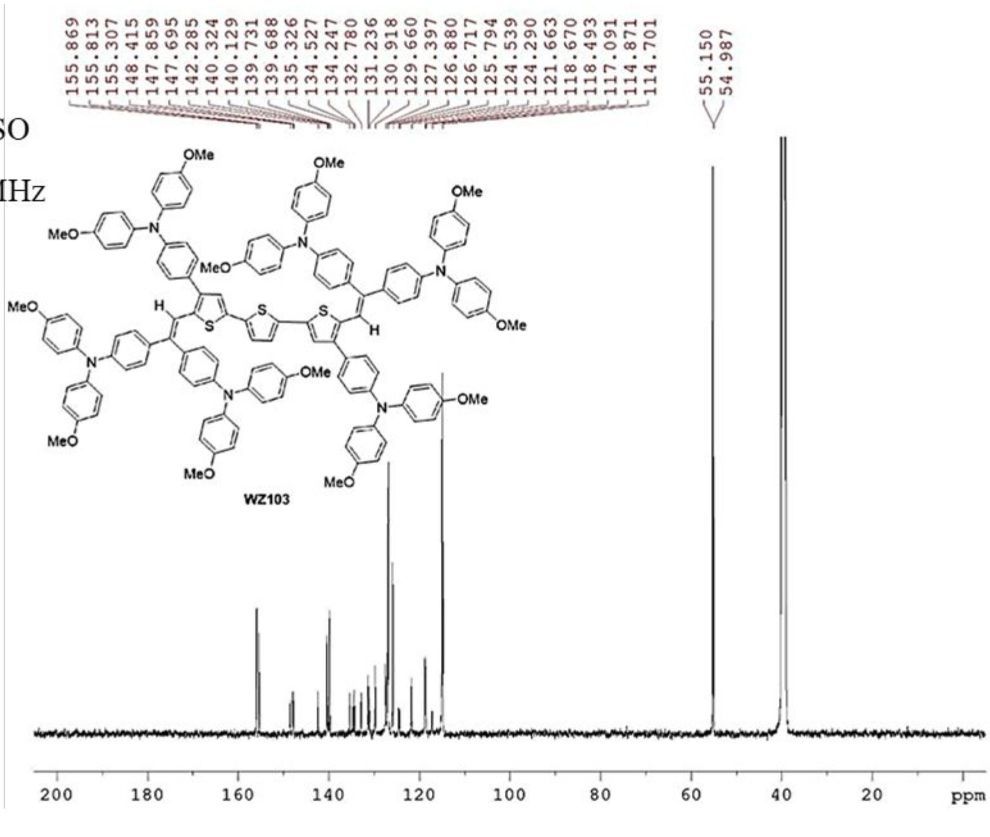

References

1. Li, H.; Fu, K.; Hagfeldt, A.; Gratzel, M.; Mhaisalkar, S. G.; Grimsdale, A. C., A simple 3,4-ethylenedioxythiophene based hole-transporting material for perovskite solar cells. *Angew. Chem. Int. Ed.* **2014,** *53*, 4085-8.
2. Li, H.; Fu, K.; Boix, P. P.; Wong, L. H.; Hagfeldt, A.; Grätzel, M.; Mhaisalkar, S. G.; Grimsdale, A. C., Hole-Transporting Small Molecules Based on Thiophene Cores for High Efficiency Perovskite Solar Cells. *ChemSusChem* **2014,** *7*, 3420-3425.
3. Liu, X.; Kong, F.; Ghadari, R.; Jin, S.; Chen, W.; Yu, T.; Hayat, T.; Alsaedi, A.; Guo, F.; Tan, Z. a.; Chen, J.; Dai, S., Thiophene–Arylamine Hole-Transporting Materials in Perovskite Solar Cells: Substitution Position Effect. *Energy Technol.* **2017,** *5*, 1788-1794.
4. Krishnamoorthy, T.; Kunwu, F.; Boix, P. P.; Li, H.; Koh, T. M.; Leong, W. L.; Powar, S.; Grimsdale, A.; Grätzel, M.; Mathews, N.; Mhaisalkar, S. G., A swivel-cruciform thiophene based hole-transporting material for efficient perovskite solar cells. *J. Mater. Chem. A* **2014,** *2*, 6305-6309.
5. Lu, K.-M.; Lee, K.-M.; Lai, C.-H.; Ting, C.-C.; Liu, C.-Y., One-pot synthesis of D–π–D–π–D type hole-transporting materials for perovskite solar cells by sequential C–H (hetero)arylations. *Chem. Commun.* **2018,** *54*, 11495-11498.
6. Zhang, F.; Wang, Z.; Zhu, H.; Pellet, N.; Luo, J.; Yi, C.; Liu, X.; Liu, H.; Wang, S.; Li, X.; Xiao, Y.; Zakeeruddin, S. M.; Bi, D.; Grätzel, M., Over 20% PCE perovskite solar cells with superior stability achieved by novel and low-cost hole-transporting materials. *Nano Energy* **2017,** *41*, 469-475.
7. Joseph, V.; Sutanto, A. A.; Igci, C.; Syzgantseva, O. A.; Jankauskas, V.; Rakstys, K.; Queloz, V. I. E.; Kanda, H.; Huang, P.-Y.; Ni, J.-S.; Kinge, S.; Chen, M.-C.; Nazeeruddin, M. K., Stable Perovskite Solar Cells Using Molecularly Engineered Functionalized Oligothiophenes as Low-Cost Hole-Transporting Materials. *Small* **2021,** *17*, 2100783.
8. Malinauskas, T.; Saliba, M.; Matsui, T.; Daskeviciene, M.; Urnikaite, S.; Gratia, P.; Send, R.; Wonneberger, H.; Bruder, I.; Graetzel, M.; Getautis, V.; Nazeeruddin, M. K., Branched methoxydiphenylamine-substituted fluorene derivatives as hole transporting materials for high-performance perovskite solar cells. *Energy Environ. Sci.* **2016,** *9*, 1681-1686.
9. Wu, Y.; Wang, Z.; Liang, M.; Cheng, H.; Li, M.; Liu, L.; Wang, B.; Wu, J.; Prasad Ghimire, R.; Wang, X.; Sun, Z.; Xue, S.; Qiao, Q., Influence of Nonfused Cores on the Photovoltaic Performance of Linear Triphenylamine-Based Hole-Transporting Materials for Perovskite Solar Cells. *ACS Appl. Mater. Interfaces* **2018,** *10*, 17883-17895.
10. Rojas, D. E. M.; Cho, K. T.; Zhang, Y.; Urbani, M.; Tabet, N.; de la Torre, G.; Nazeeruddin, M. K.; Torres, T., Tetrathienoanthracene and Tetrathienylbenzene Derivatives as Hole-Transporting Materials for Perovskite Solar Cell. *Adv. Energy Mater.* **2018,** *8*, 1800681.
11. Zhang, F.; Wang, Z.; Zhu, H.; Wang, S.; Li, X., A low-cost thiophene-based hole transport material for efficient and stable perovskite solar cells. *Org. Electron.* **2019,** *71*, 194-198.
12. Petrus, M. L.; Bein, T.; Dingemans, T. J.; Docampo, P., A low cost azomethine-based hole transporting material for perovskite photovoltaics. *J. Mater. Chem. A* **2015,** *3*, 12159-12162.

13. Li, E.; Li, W.; Li, L.; Zhang, H.; Shen, C.; Wu, Z.; Zhang, W.; Xu, X.; Tian, H.; Zhu, W.-H.; Wu, Y., Efficient p-i-n structured perovskite solar cells employing low-cost and highly reproducible oligomers as hole transporting materials. *Sci. China Chem.* **2019,** *62*, 767-774.
